# Dry Pet Food Flavor Enhancers and Their Impact on Palatability: A Review

**DOI:** 10.3390/foods10112599

**Published:** 2021-10-27

**Authors:** Shilpa S. Samant, Philip Glen Crandall, Sara E. Jarma Arroyo, Han-Seok Seo

**Affiliations:** Department of Food Science, University of Arkansas, 2650 North Young Avenue, Fayetteville, AR 72704, USA; sssamant@uark.edu (S.S.S.); crandal@uark.edu (P.G.C.); sejarmaa@uark.edu (S.E.J.A.)

**Keywords:** pet, pet food, dry dog food, palatants, palatability, flavor enhancer

## Abstract

Pet foods are a vital component of the global food industry. Pet food’s success depends on its acceptance by both consumers (the pets) and purchasers (the pet owners). Palatability tests using panels of both trained and untrained pets are often used to measure the preference and acceptability of pet foods. Human perception of pet foods is usually determined by descriptive sensory analysis. Since dry pet foods (also known as kibbles), while being the most popular, are the least palatable, palatants as a flavor enhancer are generally added to dry pet foods to increase their acceptability to pets. Pet foods can also be prepared to be more appealing to pet owners if the chosen aromas and flavors are commonly associated with human food. With increasing demand, developing flavor enhancers to meet the needs of both pets and owners is becoming increasingly important. This review summarized the current state of flavor enhancers used in the pet food industry and their influence on food palatability from both animal and human standpoints.

## 1. Introduction

The pet food industry is an expanding part of the food industry that is experiencing significant growth and has great potential for continual growth. According to the American Pet Products Association (APPA), more than $100 billion were spent on pet-related sales in the U.S. in 2020, of which $29.6 billion were spent on food alone, and the rest included supplies/over-the-counter medicines, vet care, live animal purchases, and other services (e.g., grooming and boarding) [1]. Pet food sales are forecast to be level at $33.5 billion by 2025 in the U.S. market [2] as pet ownership continues to grow. For example, in 1988, 56% of households in the U.S. owned a pet compared to 70% of U.S. households in 2021 [1]. Among many different types of pets, this review specifically focused on dogs because dogs are the most popular pets or “companion animals” in the U.S., with 69.0 million households owning dogs [1].

Pet foods are generally available in three forms: moist, semi-moist, and dry, depending on their final moisture content [3]. The dry pet food category remains the number one choice for pet owners with $5338.2 million multi-outlet sales in the U.S. in 2020, followed by the outgrowing semi-moist and moist subsegments with $2027.9 million [2]. Moist pet foods typically contain proteinaceous materials (e.g., meat, meat by-products, or fish) and have a final moisture content of 65% or more [3]. Moist pet foods have a limited refrigerated shelf life after opening. Secondly, semi-moist pet foods are prepared using a combination of proteinaceous and farinaceous ingredients (e.g., wheat, oats, or other cereal grains) with a final moisture content between 20 and 65%. Finally, dry pet foods with less than 20% moisture content are generally prepared using primarily farinaceous ingredients along with a small proportion of proteinaceous materials. Dry pet foods with a moisture content of 8–9% usually have a dry and crunchy texture, while other formulations with moisture content between 10–15% have a softer texture [4]. Dry pet foods include baked, pelleted, and extruded foods, with extruded foods the most common. Raw materials used to make pet foods generally include grains, meat, poultry, eggs, and vegetable by-products along with added fats, vitamins, and minerals [4]. Dry pet foods have a long shelf-life because of their low water activity (<0.60 aw) and thus microbial stability. However, dry pet foods generally are less attractive to pets than moist or semi-moist pet foods, probably because of their lower flavor appeal [5,6], while some pets may prefer dry pet foods because of their textural characteristics.

Since dry pet foods are most popularly used and constitute a major portion of the pet food market among the three types of commercially available pet foods [7], enhancing their palatability for greater acceptance among pets is extremely important and has been rigorously investigated. Previous research suggests that odors might be the primary drivers in a dog’s food choice [8,9,10,11,12]. For example, in a study where dogs were trained to discriminate between foods with different meat sources, Houpt et al. [11] found that dogs did not maintain discriminatory ability once they were subjected to reversible peripheral anosmia that resulted in temporary loss of their sense of smell. Olfactory cues from pet foods, constituting a major aspect of overall flavor characteristics, are important drivers of liking for pets [8], and such cues also can play an important role in influencing acceptance of the product from a pet owner’s perspective. For example, Di Donfrancesco et al. [13] found that pet owners do not appreciate pet foods with aromas that are perceived as being too strong or intense off-odor notes such as aldehydes like canaveron, oxidized oil, or must/dust, even though these odors may be attractive to the pets. In contrast, pet owners were found to like pet foods with grainy-type aromas but did not prefer other aroma attributes [13]. Pet owners’ emotions are also influenced by the sensory characteristics of pet foods. In a study by Delime et al. [14], American and French pet owners could discriminate the dog and cat dry kibbles by emotions evoked by their odors. For example, the odors “spicy”, “herbs-like”, “yeast-bouillon-like”, and “roasted chicken-like” were more associated with activation-related emotions, while the odors “fatty-rancid”, “viscera like”, and “cereal-like” were more associated with de-activation-related emotions [14]. Since food-evoked emotions have been found to play an important role in consumer acceptability [15,16] and purchasing-related behavior [17], the pet food industry should also consider pet owners’ emotional responses to pet foods. Additionally, the pet food industry is continually exploring ways to decrease objectional odors capable of being detected by pet owners while increasing desirable flavor characteristics from a pet’s perspective.

Adding certain chemical compounds to pet foods to enhance flavor characteristics is one way of increasing palatability. Since some chemical compounds can also work as masking agents, they can be added with an intention to overcome or mask off-flavors or odors, increasing human-perceived palatability. However, while many “flavor enhancers” (also known as “palatants”) have been proposed over the years, they are not always backed by thorough research to understand their acceptability among pets and pet owners. Therefore, this review focuses on two points: 1) palatability of dry pet foods from the consumer (pet) and purchaser (pet owner) standpoints and 2) flavor enhancers used as palatants in the pet food industry, especially in the category of dry dog foods or kibble, and their impact on palatability.

## 2. Palatability of Dry Pet Food

### 2.1. Pets, Pet Owners, and Palatability

Araujo and Milgram [18] characterized the “palatability” of pet foods as “*a measure of subjective food preference and depends on taste, texture, and odor*”. In addition, the palatability of pet foods has also been defined as “*pleasantness of taste of feed to animals that is understood through the sensory characteristics of food, such as taste, flavor and mouthfeel*” [19]. When developing new products of pet foods, manufacturers must achieve a balance between nutrition quality and sensory appeal because pet foods with the high nutritional quality might not be consumed if they have low sensory appeal to pets, resulting in a low repeat purchase intention by pet owners. Conversely, if pet foods are “appealing” to pet owners, they tend to generally be purchased for their pets [13,20]. Di Donfrancesco et al. [13] asked pet owners to evaluate eight varieties of dry dog food and rate both how much they liked each of them and how much they believed their pets would like them, and the relationship between overall liking of the pet owners with their prediction of dog liking was found to be very high (Pearson’s correlation coefficient = 0.93). In other words, the more a product was liked by pet owners, the higher they ultimately perceived it would be liked by their pet, leading to higher purchase intention. Furthermore, appearance, color, and aroma likings were the main contributors to the overall liking for pet owners, while product color intensity (especially darker brown colors) and oily appearance of the kibble produced a negative reaction by pet owners. Kibble size also influenced product liking, with products where kibbles were perceived as too small showing the lowest liking scores. Among the different sensory attributes, both aroma and appearance attributes have been found to be the primary drivers of pet food acceptability from the pet owner’s standpoint. From the pet standpoint, even though aromas (i.e., orthonasal odors) may drive initial preference, consumption of the product is dictated more by its overall flavors (i.e., retronasal odors) [13,21].

Palatability considerations for dogs tend to be driven by their ancestral history. Since dogs, the first domesticated animal, are direct descendants of ancient wolves [22], they tend to prefer meat-based diets over cereal-based ones [23]. Lohse [5] suggested that dogs prefer beef the most, followed by lamb and chicken, and they also prefer moist or semi-moist foods over dry ones due to the low flavor appeal of dry dog foods. Another study by Houpt and Hintz [12] found that dogs exhibit similar preferences for beef and pork, which is higher than those for chicken and lamb. While pork and beef meat ingredients, that tend to impart distinct and strong (“meat-based” or “meaty”) aromas, could be liked by pets, they might constitute a turn-off for pet owners since research has shown that pet owners dislike pet foods with excessively strong aromas [13]. In addition to the liking of “meaty” flavors, dogs have also shown a high preference for sweet-tasting substances [24]. For example, beagles aged two to four months were found to prefer sweet-tasting substances such as lactose, fructose, and sucrose, while exhibiting indifference to or rejection of maltose-based substances. Moreover, sweet-tasting substances such as sucrose were found to increase food intake and induce food selection [25]. However, influences of other factors on the palatability of pet food, such as pet breed, gender, weight, relationship to owners, taste, and olfactory sensitivity [6], should also be considered.

### 2.2. Methodologies for Measuring Palatability of Dry Pet Food

#### 2.2.1. Human (Pet Owners) Sensory Analysis

Due to the inherent difficulties of animal-human communication, it is a challenge for researchers to understand the acceptance and preference of pet foods from the animal standpoint. Although indirect methods have been developed to deduce this information (see Section 2.2.2.), pet food manufacturers rely heavily on pet owners’ perceptions with respect to the palatability of a formulation by their pets. However, this is not a simple task because of differences in the pet owner’s perception with respect to what their pets may like or dislike [19]. In addition, because the pet owners might have safety concerns or other factors making them reluctant to consume pet food samples, most research with pet owners has been focused on aroma and appearance rather than taste and flavor [13,26]. It is notable that commercially-available pet foods are regulated under the Federal Food, Drug, and Cosmetic Act (FFDCA) as follows: “*requires that all animal foods, like human foods, be safe to eat, produced under sanitary conditions, contain no harmful substances and be truthfully labeled*” [27]. In addition, the Food Safety Modernization Act (FSMA), signed into law in 2011, requires animal food, including pets, to be processed under the same good manufacturing practices (CGMPs) and hazard analysis and risk-based preventive controls that are required for human food. This rule prevents safety hazards that could potentially impact humans since pet foods are typically stored next to human food.

Given all these subjective judgments and the potential profits in making a product formulation appealing to both pets and pet owners, sensory researchers in the field of food science believe that descriptive sensory analysis could be a better method of accessing the sensory quality of pet foods. Descriptive sensory analysis techniques involve describing the product in terms of appearance, odor, flavor, taste, and texture as well as with corresponding numerical quantification. However, such analysis is usually performed by a panel of trained experts, not by general pet owners, and such a panel would typically undergo extensive training with a variety of pet foods to detect subtle product differences [19]. Using descriptive sensory analysis to understand sensory attributes of pet foods is a relatively new approach, with the results obtained from descriptive sensory analysis giving an idea as to “pleasant” and “unpleasant” attributes when they are connected to overall pet-food palatability data. Di Donfrancesco et al. [28] used 21 commercially available pet food samples to develop a lexicon for describing sensory attributes such as appearance, aroma, flavor, and texture. A total of 72 sensory attributes, reflecting the highly blended nature of the pet food products, were selected to describe the sensory attributes of dry pet foods. Some of the common aroma attributes associated with pet foods were “barnyard”, “brothy”, “brown”, “grain”, “vitamin”, “oxidized oil”, “cardboard”, and “stale”. Some aroma attributes unique to only certain products were “liver”, “fish”, “burnt”, “spice brown”, “garlic”, “celery”, “clove”, and “smoky”.

While understanding the sensory attributes of pet foods is extremely helpful, their relationship to the acceptance of pet foods among pet owners is equally important. Only a few studies have been done using traditional consumer testing methods to explore the sensory acceptance of pet foods. Among studies that have been done in this regard, several ones have involved collective perceptions of the general pet owner population [13,26]. Di Donfrancesco et al. [13] explored the relationships among descriptive sensory attributes associated with eight dry pet food products and evaluated using a trained panel and consumer acceptance as evaluated by pet owners. The results suggested that appearance even more than aroma influenced pet owners’ acceptance of the products. In terms of aroma, as mentioned in the Introduction Section, pet owners did not like products with excessively strong aromas or off-flavors such as “oxidized oil” or “musty/dusty”, while “meaty” aromas were generally acceptable to pet owners. This information provides direction to pet food product manufacturers to aim at avoiding certain pet-food attributes while including others. However, when modifying the pet foods to be acceptable to the pet owners, precautions must be taken because pets’ liking for the food cannot be compromised.

#### 2.2.2. Animal Palatability Testing (Pets)

Methods for measuring palatability of pet foods employ one of two types of panels: an expert panel (trained dogs at pet centers) or an untrained panel (in-home or family-owned pets), and both types of panels pose particular advantages and disadvantages [29,30]. Similar to the human expert panel described earlier, an expert panel consisting of extensively trained animals tends to be more accurate and reliable compared to an untrained panel of pets. In general, since palatability tests with untrained panels are less controlled and subject to increased variations based on each individual pet’s historical background, testing with untrained pets usually requires a larger number of animals (~100) compared to tests conducted with a panel of fewer trained animals (~30) [29]. However, an advantage of using an in-home panel over a trained panel is obtaining results in a “natural setting” perception of the pets while also including the owner’s reactions and acceptance. Griffin et al. [30] evaluated the extent to which the food preferences of trained dogs are representative of those reported for in-home dogs. In summarizing the findings in the literature, the authors found that in-home panels were more stable in their preferences but that expert panels were better at discriminating small differences. It was also found that the food preferences were more consistent between the different panels when testing wet products compared to dry or semi-moist products. Such differences can be explained by the pets’ level of training, their feeding history, and/or the testing environment condition.

Testing methods developed for dogs tap into their inherent feeding behaviors as mentioned in Aldrich and Koppel [31]: “*dogs are opportunists for meals and will augment their diet with foraging for anything seemingly edible from other animals’ feces or scat, to insects, berries, and grass… In the dogs’ mouth are large canines, small premolars, and they lubricate their food with a serous saliva, sans amylase, produced from four primary salivary glands located throughout the orthonasal cavity. Not much time is given to masticating and savoring the food during the eating bout: Dogs are known to devour the food in a gluttonous manner and then regurgitate and re-consume at a time and place when away from competing mouths*”. Broadly, two classes of pet food palatability tests have been suggested: consumption and non-consumption tests; the former being more popular [31,32]. Classical consumption tests assessing preference and acceptance of pet food are known as “two-pan” (also called as “two-bowl”, “paired stimulus”, or “versus”) test and “one-pan” (also called as “one-bowl”, “single stimulus”, or “monadic”) test, respectively [29,31]. In the two-pan test for preference, two test foods (e.g., foods A and B) are simultaneously presented to a dog in identical pre-weighed pans and test ingredients. Dogs have free access to the pans for the period of feeding (15–30 min) after which the pans minus test ingredients eaten are again weighed. Sometimes the process is repeated for each animal while switching pan position to control for position bias [29]. Differences in weight correspond to the quantity of a test food consumed and are interpreted as a preference for that food [26]. For example, if more of food A is consumed than food B, food A is considered to be preferred over food B. This can be expressed as an intake ratio (IR) (of food A) defined as a proportion of food A consumed over the sum of food A and food B consumed. The consumption ratio (CR) (for food A) is defined as a proportion of food A consumed over food B consumed. Another important parameter assessed using a two-pan test is the first choice, i.e., the food first approached by the animal, an indication of visual and odor attractiveness of the food. Two-pan tests are typically performed with a trained animal panel [29,33]. Although two-pan tests have been proven reliable and extensively used in palatability studies, they tend not to be sensitive to long-term satiating effects of food while accounting for nutritional and caloric value. There is also a possibility, especially for those not trained to self-limit their food intake, that animals could consume excessive food [18,29]. Since the preference measured in this test is only relative between the two foods tested, each combination of pairs must be evaluated for preference when three food products or more are compared [31]. In addition, when the two products tested showed no difference with respect to intake ratios and first choice, it is difficult to conclude that the dogs preferred the two products equally because of their similar odor preference because the dogs might have a lack of olfactory discrimination [8]. Therefore, Basque et al. [8] suggested a complementary approach, i.e., a combination of food preference tests and food olfactory discrimination tasks, for providing a better understanding of the drivers of dogs’ preference for food products. Another major limitation of such tests is that, even though the “first choice” parameter reflects olfactory attractiveness, it does not truly discriminate among visual attractiveness, odor intensity, individual odor preference, and recognition due to previous experience [34]. Recently, Pétel et al. [34] developed false-bottom bowls (FBBs) intended to hold different odor compounds that modify traditional bowls by adding a drilled, stainless-steel separation plate at the bottom. Using such bowls, pet preference could be evaluated while varying aromas and measuring the role of odor acceptance among pets. However, other limitations such as the proximity of the two stimuli, i.e., test and treatment, during the evaluation have not been addressed yet.

Consumption methods also include acceptance testing using a one-pan test. In this method, the animal has free access to a single food for a given feeding period. Parameters measured include quantity consumed, speed of consumption, or enthusiasm with respect to eating the food [26,29,31]. Since these parameters are similar to those observed by a pet owner while introducing a new food product to their pet, one-pan tests are most suitable for family-owned, untrained animal panels [35]. Unless pet food samples are extremely aversive and therefore not consumed by the pets at all, their acceptability can broadly be measured with respect to food consumption required to maintain calorie intake without necessarily evaluating their taste or aroma attributes [31].

Due to limitations of consumption tests, some researchers have used facial expressions of pets as indicators of food acceptance or rejection [36], although this approach has not gained widespread use. Non-consumption tests, in which pets are trained to associate their food preferences with objects, tapping their cognitive ability, and thereby validating discriminability while assessing preference or acceptance, are also being explored. These methods tend to ensure more robust results and are seemingly not as biased by testing conditions or pet history [18]. Recently, Cheli et al. [37] suggested the use of electronic nose (e-nose) or tongue (e-tongue) as rapid tools for identifying key odorous or tasting compounds to ensure high nutritional properties of the pet food along with meeting palatability standards. While such techniques could help discriminate between different aromas/tastes and hopefully replace traditional animal-preference tests, the use of e-nose and e-tongue in the pet food industry is still new and requires more validation before being used commercially.

## 3. Overview of Palatants Used in Dry Pet Foods

Palatants, or flavor enhancers, applied to pet foods to improve their inherent palatability and increase pet acceptability, were originally referred to as “digests”, essentially proteins broken down enzymatically to provide a sensory impact of meat flavors [3,38]. These palatants can be characterized as complex systems that consist of a variety of macros and micro-molecules improving the sensory experiences of the pets and pet-owners, masking unpleasant tastes and off-flavors, and enhancing appetite in pets. [3,38]. Meat-based (e.g., poultry, pork, beef, or fish) and vegetable-based (e.g., corn, soy, potato, or grains) palatants include components such as proteins, yeasts, phosphates, antioxidants, antimicrobials, and processing agents. Essential oils, aldehydes, and condiments could also be used as palatants [39].

When designing palatants, it is important to consider their inherent properties and their interaction with the chemical composition of a pet food matrix. Commercially-available palatants, used as flavoring agents, are most often classified as either dry powders (generally added in amounts between 0.5% and 2%) or liquids (generally added in amounts between 1% and 3%) and are most commonly sprayed onto dry food, although a few could be added as an ingredient during processing. There are, however, challenges associated with proposing palatants for commercial and industrial use. First, since food palatability differs with the food form, palatants used in canned or semi-moist pet foods might not be useful for dry pet foods. Second, because palatability might differ among different species, palatants used in cat foods do not generally serve a similar purpose in dog foods. Finally, it must be ensured that the palatants used do not compromise or impact the digestibility of the food [7,20].

Extensive research has led to the discovery of some traditional palatants that have been used in the pet food industry for a long time. A number of patents have been filed claiming palatability improvement of pet foods using traditional palatants, such as amino acids [40,41], fat or fatty acids [42], and animal digests from beef, pork, poultry, or fish, etc. Organic acids such as phosphoric acid, citric acid, tartaric acid, fumaric acid, lactic acid, acetic acid, and formic acid have also been used in the past to improve the palatability of pet foods [43]. Phosphates, pyrophosphates, and polyphosphates have also been explored as potential palatants in dry pet foods [44], although there are some concerns regarding their long-term effects on renal functions of pets [45,46,47]. Such palatants could be added either as an ingredient during a mixing process or as a surface coating after product processing. A combination of different palatants is often used. For example, U.S. Patent No. 5,186,964 to Gierhart and Hogan [48] revealed the palatability-enhancing effects of phosphate, pyrophosphate, and polyphosphate when optionally combined with organic acid (e.g., citric, tartaric, fumaric, lactic, acetic, formic, or hexamic acid) and flavorants. In that case, a two-step coating process was adopted wherein the flavorant and phosphate are initially applied to the pet food, followed by spraying of an acidic enhancer. When patents mention the use of chemical compounds as potential palatants, it is worth noting that other factors such as the inherent properties of pet food and the method of palatant application can also impact an increase in palatability.

While it can be recalled that palatants can serve the purposes of improving flavor [49,50], odor [50], texture [44], appearance [26], or a combination of these attributes to increase overall preference and acceptance of a product, it is not always straightforward to determine exactly which sensory attribute is being targeted by a palatant. In a study using multiple-coating techniques, dry pet foods were coated with a primary coating comprised of farinaceous and proteinaceous material to impart the desired flavor and texture, followed by a secondary coating of starch or egg to provide the final product with a glistening sheen to make it more attractive [51]. Pyrophosphates, in particular sodium tripolyphosphate, have been used as palatants for improving the texture of the product. They provide high pH, high ionic strength, and higher protein solubility in meat, resulting in firmer texture [44]. Similarly, it is possible that animal digests provide distinct “meaty” notes to pet foods, thereby increasing palatability for dogs. U.S. Patent No. 9,480,275 B2 to Brent [52] aimed to improve palatability by making products more attractive to consumers. Specifically, the patent states “*the present invention enhances ease of access and manageability by a pet with improved attractiveness to a consumer and provides improved texture and palatability”.* Even though different palatants may work to improve different attributes of the pet food, alone or in combination, it appears that palatants that improve flavor quality of the pet food are most popular, possibly because odor/flavor characteristics are primary drivers of pet food preference and acceptance. In addition, pet owners’ attitudes toward pet foods are also modulated by the aromas and flavor attributes of the products. For example, previous research suggests that pet owners might have a negative attitude toward pet foods if the foods have strong and objectional aromas such as “musty” or “oxidized oils” [13,53]. Moreover, although strong “meaty” aromas may not be well-accepted by pet owners, they might actually be desirable to a pet. Therefore, the most desirable flavor enhancers would increase the flavorful-ness of dry pet foods for pets while also being acceptable to pet owners as mentioned in U.S. Patent No. 2015/0056347 A1 [54]: “*many pet foods proposed so far have a major disadvantage due to the presence of smells that are not appealing to the pet owners. Reciprocally, food products that are attractive to pet owners are not systematically palatable to pets.”* Therefore, the off odors must be either masked or overpowered by more flavorful characteristics, a purpose sometimes served flavor enhancers used as palatants.

## 4. Flavor Enhancers Used as Palatants and Their Impact on Dry Pet Food Palatability

### 4.1. Animal Digest and Other By-Products

Animal digest, i.e., partially hydrolyzed animal parts in both dry and liquid forms, is probably the most commonly used flavor enhancer in the pet industry. In fact, AAFCO includes animal digest as an ingredient in pet foods and defines it as “*a material which results from chemical and/or enzymatic hydrolysis of clean and undecomposed animal tissue. The animal tissues shall be exclusive of hair, horns, teeth, hooves, and feathers, except in such trace amounts as might occur unavoidably in good factory practice and shall be suitable for animal feed. If it bears a name descriptive of its kind or flavor(s), it must correspond thereto”* (p. 360, [3]). For pork and beef, non-rendered clean parts such as lungs, spleen, kidneys, brains, livers, blood, stomachs, and intestines are used. For poultry parts such as livers, hearts, heads, feet, and viscera are used. In some ways, animal digest serves as a primary palatant in pet foods because it provides raw or meaty odor/flavor notes to pet foods, making them desirable to the pets. The enzymes used in this process are generally proteases and lipases that break down proteins and fats, respectively. Additionally, the fatty acids and amino acids produced as a result of reacting with reducing sugars producing flavorful aromatic substances via the Maillard reaction are characterized to have aromas of “meaty” and “brothy” notes in the descriptive sensory analysis by a trained human panel [13]. U.S. Patent No. 4,211,797 to Cante et al. [55] disclosed the palatability-enhancing potential of beef digest along with lipolyzed beef tallow added at 4–8% by weight of dry pet food in the form of a surface coating. Consumption tests showed that the dogs preferred food with a digest of beef and beef tallow coating over control pet food not containing a palatant. Researchers have also explored the addition of protein hydrolysates (proteins hydrolyzed into short peptides and certain amino acids) directly to pet foods as flavor-enhancing palatants, and both seafood and animal protein hydrolysates have been investigated for this purpose [56,57]. U.S. Patent No. 2008/0280274 A1 to Friesen and Yamka [58] disclosed the use of poultry-liver hydrolysate either alone or in combination with poultry fat to increase the palatability of dry pet foods. Palatability was evaluated using a two-pan test with 25 dogs revealed a higher preference for food with liver hydrolysate than for pet food without it. The current research focused on animal digest as a palatant is related to optimizing the processing conditions of enzymatic treatment or using different sources of proteins and fats.

### 4.2. Maillard Reaction Precursors and Products

The Maillard reaction plays a pivotal role in flavoring pet foods. The initial stages of the Maillard reaction involve condensation of the carbonyl group (reducing sugar) with an amino compound (amino acids) by the impact of high temperatures, or at a slower rate, by low temperatures, low pH, and low a_w_ levels [59]. The condensation products are further degraded to produce different oxygenated compounds that interact with other reactive compounds such as amines, ammonia, hydrogen sulfide, and aldehydes, leading to the production of flavor compounds such as furans, pyrazines, esters, thiopenes, and other heterocyclic compounds [60]. As mentioned above, amino acids produced by animal digest react with reducing sugars to produce Maillard reaction-related flavors.

Another approach to initiating a Maillard reaction to produce flavorful compounds is to treat farinaceous ingredients with different enzymes to produce simple sugars that can react with proteins in the food system. U.S. Patent No. 6,926,917 B2 to Parthasarathy [61] proposed contacting the raw ingredients (including farinaceous together with some proteinaceous compounds) with α-amylase from 0.05% to 0.5% by weight of ingredients. The enzyme breaks down starch and other complex carbohydrates in the farinaceous ingredients into sugars, with reducing carbonyl groups serving as precursors of the Maillard reaction. The enzyme could either be added on the surface as a coating or added into the ingredients before extrusion. The inventors did not include palatability testing in their patent, but they ensured that the inclusion of α-amylase prior to extrusion improved the palatability and texture of dry pet food. Additionally, U.S. Patent No. 3,617,300 to Borochoff et al. [62] indicated that dextrose, a reducing sugar, has the potential to be used as a flavor-enhancing compound. According to this patent, some of the starch content in the dry pet foods can be converted to glucose by α-amylase and amyloglucosidase. In addition, U.S. Patent No. 4,393,085 to Spradlin et al. [63] revealed that palatability of dog foods could be improved when the slurry mixture, combined a portion of farinaceous ingredients (e.g., wheat and corn) treated with amylase and a proteinaceous portion (e.g., meat meal and soybean meal) treated with protease, was sprayed as a coating on dry dog foods. Consumption tests with 21 dogs showed that food treated with palatants developed as a combination of proteinaceous and farinaceous ingredients was preferred by the dogs over food treated with palatants containing only proteinaceous or only farinaceous ingredients. It is important to highlight that Spradlin et al. [63] did not indicate the type of pet panel (i.e., trained or untrained) that they employed in their palatability testing. Similar approaches in modifying processing conditions to treat proteinaceous and farinaceous raw materials in the production of pet foods were found to contribute to enhancing the flavor profile of the final product [64]. While the approaches discussed in this section have been focused on initiating the Maillard reaction to produce flavor compounds, some researchers have explored the direct addition of Maillard reaction-precursors such as amino acids to the pet foods, as explained below (Section 4.3.).

The final flavor compounds produced during the Maillard reaction can be directly used as flavor additives. Recently, Chen et al. [65] investigated the optimization of key aroma compounds, referred to as “dog food attractants” (DFAs), in pet food. In their study, seven DFAs, based on the Maillard reaction products using protein sources (brewer’s yeast, chicken meal, and soybean meal) and reducing sugar (xylose), were identified. Headspace-solid phase microextraction (HS-SPME) and gas chromatography revealed 53 aroma compounds associated with these seven DFAs, including 11 aldehydes, 10 heterocycle compounds, seven alcohols, seven esters, four ketones, four organic acids, four phenols, two terpenes, and two polycyclic aromatic hydrocarbons. Alcohols are generally characterized by fruity odors, while some aldehydes such as benzaldehyde smell like almond oil [65,66]. Benzaldehyde and hexanal (green or rose-like odors) are common aldehydes associated with pet foods [67]. Another reactant compound of the Maillard reaction, furfural, imparts a caramel-like flavor to pet foods [65]. In fact, caramel-like sweet odors have been found to be liked by dogs [68]. For further investigation, Chen et al. [65] performed a preference (two-pan) test and an acceptance (one-pan) test with eight beagle dogs. For the acceptance test, the intake ratio (IR) was calculated for each food, while for the preference test, first choice and first-approach data were used to measure preference for a sample. The results showed that 2,5-dimethyl pyrazine, vanillin, and benzaldehyde, added at levels of 0.21, 8.90, and 1.82, µg/g of dry food, respectively, were found to make a significant contribution to enhanced palatability of the DFAs. The advantage of using these aroma compounds as flavor enhancers is that, along with being attractive to dogs, they are also deemed pleasant by pet owners. However, it is worth mentioning that while the Maillard reaction is an important chemical reaction for pet food manufacturers to increase pet acceptability of the food, there are some concerns about Maillard reaction products reducing the bioavailability of lysine resulting in reduced nutritive value of the pet foods. Some Maillard reaction products have also been associated with age-related diseases in pets [69,70].

### 4.3. Amino Acids

Amino acid lysine in addition to other amino acids such as tyrosine, arginine, and tryptophan have been popularly explored as a flavor enhancer in human foods [71] as well as in pet foods. In U.S. Patent No. 4,267,195 to Boudreau and White [40], it was disclosed that a coating of L-lysine on the surface of dry dog foods at a ratio of 0.1 to 500 mM had the potential to increase the palatability of pet foods while meeting the nutritional requirements of dogs in terms of adequate proteins, carbohydrates, vitamins, and minerals. Although palatability testing with dogs is not reported in this study, the authors of this patent described that *“test have shown all of the dog food flavors of this invention to cause strong taste responses in dogs”.* Another patent, U.S. Patent No. 4,282,254 to Franzen et al. [41] revealed the flavor-enhancing potential of L-phenylalanine, L-tyrosine, L-methionine, L-tryptophan, L-arginine, L-leucine, L-isoleucine, L-serine, and combinations of them in an amount of between 0.001% and 0.8% by weight. The patent reported improvement in palatability based on palatability testing using 40 dogs in an incomplete block design. The patent also claimed that L-methionine at concentrations between 0.005% and 0.5% based on the weight of dog food had a palatability-enhancing effect in both dry and semi-moist pet foods. In addition, E.P Patent No. 2731449 B1 to Niceron [72] evaluated the palatability of dry dog foods coated with a mixture of amino acids (alanine, aspartic acid, asparagine, arginine, cysteine, glutamic acid, glycine, glutamine, histidine, isoleucine, leucine, lysine, methionine, phenylalanine, proline, serine, threonine, tryptophan, tyrosine, and valine). Based on a two-pan test using 36 dog panelists, the bowls of a control diet (no amino acids added) and the experimental diet (amino acid palatability-enhancing composition) were compared from 15 to 30 min. The patent reported that the palatability of the amino acid mixture did not differ from the control. However, the same patent demonstrated higher palatability of the amino acid diet with a cat panel, highlighting the difference in palatability between the two specifies.

### 4.4. Aroma/Flavor Compounds (Direct Addition)

Since the primary goal of flavor enhancers is to improve the flavor profile of pet foods and thereby their palatability, the addition of aroma or flavor compounds directly into pet foods seems only logical. In addition to Maillard reaction-related flavors mentioned above, other aromas have also been used for this purpose. In patent U.S. 2015/0056347 A1 [54], human sensory appeal and pet preference were both evaluated when dry pet foods were prepared with additions of different combinations of palatability enhancers and aroma compounds. Dry food aromas could include animal (beef, poultry, pork, or fish), vegetable (herbs, fruits, or vegetables), and dairy (butter, milk, or cheese) compounds. In addition to aroma compounds, traditional palatability enhancers such as animal digests, animal fats, or dairy products were used. In the total composition of aroma and palatability enhancer, the liquid aroma was varied from 0.25 to 20%, while the remainder (80–99.75%) was composed of the palatability enhancer. This was one of the few patents that reported sensory studies validating palatability-enhancing effects using both animal and human panelists. Pet preference was evaluated using a two-pan test of 36 trained dogs. The first food consumed, and the amount of each food consumed was calculated by the end of the test (from 15 to 30 min). Human panelists “trained” with pet foods were used for sensory assessment of the food samples. Each panelist was asked to rate his/her preference for the odor on a 9-point scale with respect to liking (1 = I do not like it at all; 9 = like it very much) and odor intensity (1 = not at all intense; 9 = very intense). It was claimed that adding a coating of at least one dry food aroma to at least one other palatability enhancer resulted in a flavor-enhancing effect in dry pet foods, based on both animal and human panel results.

A recent U.S. Patent No. 2016/0309749 A1 to Perez and Dodge [73], suggested coating the dry pet foods with a mixture of liquid palatability enhancer and liquid aroma followed by a coating of the animal digest to seal the aroma. In the total composition of aroma and palatability enhancer, the liquid aroma was varied from 1 to 5%, while the remainder of 95–99% was composed of the palatability enhancer. The results revealed that the use of a traditional liquid palatability enhancer along with liquid aroma used with dry pet foods increased palatability among pets and pet owners. The aromas were specifically chosen to impart the “*humanization sensory factor without a negative sensory impact for the pets that will consume the dry pet food*” [73]. Therefore, “sweet” and “grilled” liquid aromas (referred to as “human” aroma) were used in this invention. The advantage of using the liquid aroma and the liquid palatability enhancer over their dry counterparts is that it is more cost-efficient and negates the requirement for a dry aroma to be dissolved into a carrier for spraying as a surface coating. U.S. Patent No. 6,379,727 B1 [74] revealed the packaging of flavors in dry powder (in a shaker) or liquid (in a spray bottle) form along with a base dog food. The flavors explored during this work, including BBQ, beef stew and crackers, pizza, sausages and eggs, roast beef, peanut butter and jelly, and potato, are popularly found in foods for human consumption. This method, i.e., directly adding aromas and flavors to pet food, is gaining popularity although palatability testing to validate the palatability-enhancing effect is required.

### 4.5. Lipids (Fats, Oils, and Fatty Acids)

Past research suggests that the macronutrient profile of the food can influence intake among pets [75,76]. Specifically, foraging decisions made by dogs could, to some extent, be based on meeting their macronutrient goals. In a diet selection study, Hewson-Hughes [75] investigated whether dogs regulated their diets to meet certain macronutrient intake. It was found that irrespective of the breed, dogs regulated their diet to a protein: fat: carbohydrate ratio of approximately 30%: 63%: 7% by energy. Certain pet food ingredients, such as lipids, in addition to their essentiality to maintain the macronutrient profile, also function as palatants. For example, crude fat extracts, generally from sources such as chicken, beef, lamb, and other land animals, have characteristic aromas that may be desirable to pets [77]. However, the application of such crude fats is not preferred due to the presence of saturated and higher-melting temperature fats that tend to integrate together and trap the aroma molecules. Such “fatty” clusters tend to stick to the surface of the pet food, rendering it less appealing in terms of appearance. In addition, because high levels of dietary fat (e.g., >10%) may increase the potential for diet rancidity, antioxidants may be added into pet foods if the nutrient quality and quantity of the foods are to be preserved [78]. U.S. Patent No. 3,745,023 [79] used a combination of modified lower-melting temperature land-animal fat (50 parts by weight), vegetable oil (49 parts by weight), and other flavorants (1 part by weight), prepared by vigorously stirring rendered chicken, beef, and pork fat along with crude soybean oil at room temperature and cooling to 7.2 °C. The precipitated saturated fats and soybean sludge was filtered off using the filtrate for further processing. The filtrate was mixed with other flavorants such as garlic and anise oil along with some antioxidants. This composition, when sprayed over the dry pet food as a coating, was proposed as a flavor enhancer. Fish oil could also be used. Interestingly, using fats as palatants could add to the nutritional profile, act as a carrier for other flavors, and impart specific aromas and flavors. Fournier [80] tested the palatability of eleven fat types of three different origins (pork, poultry, and beef) with a two-pan test by a dog expert panel. The fat types were used as a coating at a 6% level with 1.5% of premium liquid dog palatant and tested against a control coated with 6% of poultry fat and 1,5% of the same premium liquid dog. In general, beef tallow and the mix between beef and pork were the most palatable, and pork fat was more palatable than poultry fat as shown by higher consumption rates. Noticed differences in preference among fats of the same origin (poultry or beef) were also found, suggesting an impact of fat manufacturing process and composition (volatiles and fatty acids) on palatability. In addition, U.S. Patent No. 2016/0029668A1 [81] revealed a palatant composition derived from cocoa butter or a mixture of different fatty acids. Specifically, mono-ethaloamine was exposed to heat treatment and an amidation reaction along with the fats, oils, or fatty acids, thereby enhancing the palatability of pet food when added at a level of 10–1000 ppm to the pet food. A palatability-enhancing composition prepared using a mixture of ethanomides with a fatty acid (oleic acid, palmitic acid, stearic acid, linoleic acid, or linolenic acid) was blended with the other ingredients and extruded to form dry kibble. This composition could be added as a surface coating on the extruded kibble in addition to being blended with the ingredients before extrusion. Preference for the palatant-added food over the control food was validated with a two-pan test using a dog panel, with higher consumption indicating higher preference. However, the impact of fatty acid addition on dog food’s palatability seems to be dependent on the chemical configuration of the molecules (e.g., carbon-chain length). For example, using a two-pan test, Dahkal and Aldrich [82] evaluated the palatability of diets containing medium-chain fatty acids including caproic acid (C6, 50%), caprylic acid (C8, 50%), and capric acid (C10, 50%) with a 20-dog panel. The results revealed that adding medium-chain fatty acids, to a diet strongly decreased food acceptance of dry dog foods compared to a control (chicken fat) [82].

### 4.6. Organic Acids

Organic acids have been successfully used in dry pet foods to increase palatability and provide an antimicrobial effect. The U.S. Patent No. 2014/0154356 A1 [43] proposed the use of fumaric acid, either alone or in combination with sorbic, succinic, and gallic acids as potential palatants and anti-microbial agents of dog kibbles. The palatability of the organic acids was tested using the two-pan test with a dog panel. The diets were presented for a period of four hours, and the amount consumed was recorded for each dog. The results indicated that gallic acid and fumaric acid displayed acceptable palatability for inclusion in dry dog palatant formulations compared to the control (off-the-shelf brand name). The inclusion of sorbic acid on its own or in combination with salt as a successful palatant was also demonstrated by patent U.S. 2018/0220678 A1 [83]. Palatability comparisons between dry kibbles coated with sorbic acid and a control (no sorbic acid) were carried out using a two-pan procedure with a panel of 20 dogs. The pair of bowls with weighed amounts of test products was presented to each dog for 20 min, or when the bowl was empty if sooner. The test products coated with sorbic acid showed a higher consumption rate over the control with no sorbic acid, as well as when compared to commercial dog food. A recent patent, U.S. No. 2016/0316789 A1 to Aubril and Callejon [84], disclosed ascorbic acid for potential use along with traditional palatability enhancers to improve the flavor of the pet food. A two-pan test with 36 dogs was conducted, with the overall duration of study for each dog ranging between 15–30 min. The first food consumed, and the amount of food consumed were both calculated for each pet food, although the exact role of the acid, either antimicrobial action of flavor-enhancing effect or both, was not highlighted. The palatability results showed that the addition of ascorbic acid (between 0.005% and 0.3% by weight) increased the palatability of the dry kibbles compared to the control (no ascorbic acid in the mix), regardless of the level of ascorbic acid tested.

### 4.7. Miscellaneous/Others

As mentioned earlier, novel ingredients are being explored as potential flavor-enhancing agents. One such substance is ammoniated glycyrrhizin, processed from natural sources and with 50 times more sweetness than sucrose. Glycyrrhizic acid is obtained by grinding the root *Glycyrrhiza glabra* (licorice), followed by extraction of the ground material with hot water, and recovery of the acid-insoluble fraction from the extract containing glycyrrhizic acid. Glycyrrhizic acid can further be ammoniated to provide ammoniated glycyrrhizin, known to work synergistically with sucrose, meaning that the sweetness value of glycyrrhizin and sucrose combined is more than the mere additive effect or sum of their individual sweetness values. This approach both targets taste improvement and improves overall flavor appeal [85]. The flavor-enhancing effect of ammoniated glycyrrhizin was validated with a consumption test using 20 dogs. Since dogs like the sweet taste and sweet flavors, glycyrrhizin can be used to impart the desired taste, thereby reducing the sucrose content of the pet food as well.

Herbs and spices are another class of flavoring compounds used to increase the palatability of pet foods. W.O. Patent No. 2006/065841A2 [86] exclusively calls out the impact of tarragon essential oil as a palatant. The palatability of the herb was measured with 25 dogs via a two-pan test. Test foods were dry dog foods prepared with or without the addition of essential oil of tarragon to the pre-conditioner composition during the preparation of the foods. The foods were left with the animal for 45 min, and they were reweighed at the end of testing to determine the intake ratio. The addition of tarragon oil to the dog food, especially at low concentrations (0.001–0.005 wt%), significantly increased the palatability of dry dog food compared to the control. Other spices such as garlic and anise have also been used because of their distinct odors.

Zeolites, essentially microporous, aluminosilicate minerals, have also been explored as potential palatants because of their adsorbent and catalytic properties. For example, U.S. Patent No. 2009/0274796A1 [87] disclosed the use of clinoptilolite, a zeolite, as a flavor-enhancing palatant. For dry pet foods, the palatant is sprayed on the surface of the food to form a coating. The patent reported results from a two-pan preference test using 25 dogs, wherein food with zeolite was found to be consumed more than food without the zeolite. In addition, food with zeolite was ingested by the dogs more frequently and at a higher time rate, further indicating higher preference. Barnes [88] provided an extensive market analysis of U.S. pet foods and suggested that many pet food manufacturers, in particular dog food manufacturers, use cranberries in their products for health reasons. Since cranberries are used in human food for nutrition and flavoring, there is a possibility of exploring the use of cranberries, and perhaps other berry flavors, for flavor enhancement in pet foods. Other palatants such as pyrophosphates have also been explored [44], although pyrophosphates have been shown in the past to improve textural aspects of food more than flavor [89], and the mechanism to improve the palatability of pet food is still unknown. On similar lines, U.S. Patent No. 6,926,917 B2 [61] suggested the use of α-amylase to improve the palatability of dry pet foods by means of increasing product softness. Although new chemical compounds have been suggested for their role as flavor enhancers, the exact mechanisms of their functionality are not well-known.

Table 1 shows some patents published for strengthening palatability of pet (dog) food using flavor enhancers prepared from different types of substances..

## 5. Synonymy between Human and Pet Foods: Future Directions

A growing trend in the pet food industry is to be in synchronism with the human food industry. As mentioned earlier, pet owners’ decision-making in buying food for their pets tends to be in harmony with their own food-related decisions [20]. In other words, if a pet owner is interested in “organic” and “natural” foods, it is highly likely that they prefer to see those same claims for the pet food products they purchase for their dogs. U.S. Patent No. 2015/0079042 A1 to Foley and Harper [94] disclosed a method for developing a nutritious snack in the form of dog treats made of organic and natural ingredients. All the ingredients, including wheat flour (42.4%), blueberries (15%), cinnamon (0.1%), yogurt with or without vanilla flavor (21.6%), and almond milk (21.1%), were mixed together into a dough and baked in the desired shape to form the dry pet food. This patent reflects a shift in ingredient choice and requirements by pet owners when they make pet food-related decisions. Another example is U.S. Patent No. 2006/0062892 A1 [95] that described a dry pet food for dogs using a combination of meat and vegetables. In particular, the proposed pet food is a dry kibble mixed with dried meat jerky, along with dehydrated/freeze-dried fruits/vegetables and dehydrated natural gravy. A wide selection of meat (beef, pork, chicken, duck, turkey, buffalo, fish, venison, and other seafood), vegetables (potatoes, sweet potatoes, carrots, peas, beans, zucchini, squash, green beans, hominy, corns, tomatoes, and spinach), and fruits (apples, blueberries, peaches, cranberries, cantaloupe, pears, apricots, blackberries, papaya, strawberries, mangos, and raspberries) could be used to prepare a pet food that is both nutritious and tasteful. Another invention, U.S. Patent No. 2017/0181449 A1 [96], developed a pet-food product in which the food mixture (animal-based) was positioned on a chew stick comprising of pizzle stick, giving it an appearance of shish kabob, and providing a long-lasting chewing portion. A vegetarian alternative, U.S. Patent No. 2017/0181448 A1 [97], used a plant mixture of a large variety of fruits and vegetables for the base food that was then positioned on a rawhide chew. Intriguingly, the use of probiotics to improve the intestinal microbiota of dogs has also been explored, even though studies focusing on associated palatability are still scarce [98,99]. U.S. Patent No. 8,691,303 B2 [99] disclosed a method for dusting dry pet foods with a powder containing probiotics, with the probiotic at least 10^5^ CFU/g of the kibble. It is suggested that probiotic dusting does not negatively hamper acceptance of dry pet food. It will be interesting to examine how other trends in the human food industry continue to slowly seep into the pet food industry and further increase the high correlation between them.

## 6. Conclusions

While dry pet foods are the most popular pet food category purchased by pet owners because of their long shelf life, ease of preparation, and low price, they tend to be low in terms of their palatability to dogs compared to the wet and semi-moist types. Since olfactory cues (aroma and flavor) are the primary drivers of a dog’s palatability, pet food manufacturers add certain substances (or flavor enhancers) to pet foods to improve their flavorful-ness and thereby its overall palatability by dogs. The present work provides a summary of the current palatants used in the pet food industry and their impact on the palatability of dog foods. In addition, it reviews the current methodology employed to evaluate the sensory acceptance of these palatants from both pet and pet owner standpoints. A promising approach lies in the direct addition of aromas and flavors, along with traditional palatability-enhancing agents to increase pet-food palatability. Furthermore, the employment of “humanizing” aromas, i.e., aromas commonly related to human foods (e.g., BBQ, grilled, or caramel-like aromas) could be valuable with respect to pet food acceptance when added in quantities that are both pleasant to the pet owners and do not negatively impact the pets’ acceptance of food.

## Figures and Tables

**Table 1:** List of patents published for improving palatability of pet (dog) food using flavor enhancers as palatants.

Category	Target Palatants	Amount Suggested	Palatability Testing		Patent Number	Reference
			Pet (Dog)	Pet Owner		
Animal digest and other by-products						
Beef protein and fat	Blend of beef digest and lipolyzed beef tallow	4–8% based on total weight of the dry dog food	Yes	No	U.S. 4,211,797	[55]
Vegetable protein in combination with animal fat or oil	Bleachable fancy tallow, butter oil, soy isolate, enzymes	0.1–5% based on total weight of the dry dog food	Yes	No	U.S. 3,857,968	[90]
Protein hydrolysate	Poultry liver hydrolysate (alone or in combination with poultry fat)	0.01–6% based on total weight of the dry dog food	Yes	No	U.S. 2008/0280274 A1	[58]
Maillard reaction precursors and products						
Combination of reducing sugar, animal blood, yeast and fat	Glucose, dried animal blood, yeast extract, bleachable fancy tallow	1–5% based on total weight of the dry dog food	Yes	No	U.S. 4,089,978	[91]
Sulfur compounds, reducing sugar, animal digest	Sulfur compound (ammonium sulfate, ammonium hydrogen sulfate, diammonium carbonate, ammonium bicarbonate, ammonium chloride, ammonium nitrate, ammonium hydroxide, di-ammonium phosphate and mixtures thereof), reducing sugar (xylose, pentose, glucose, fructose, starch hydrolysates, molasses, and mixtures thereof	Varies with composition of food	Yes	No	U.S. 6,660,319 B1	[92]
Digested proteinaceous and farinaceous ingredients	Soy, whey, blood plasma, egg, chicken skins, cheese, enzymes	20–80% farinaceous ingredients and 20–80% proteinaceous ingredients	Yes	No	U.S. 4,713,250	[64]
Digested proteinaceous and farinaceous ingredients	Meat and bone meal and soybean (proteinaceous), wheat corn (farinaceous), enzymes	20–80% farinaceous ingredients and 20–80% proteinaceous ingredients	Yes	No	U.S. 4,391,829	[93]
Digested proteinaceous and farinaceous ingredients	Meat and bone meal and soybean (proteinaceous), wheat corn (farinaceous), enzymes	20–80% farinaceous ingredients and 20–80% proteinaceous ingredients	Yes	No	U.S. 4,393,085	[63]
Digested proteinaceous and farinaceous ingredients and α-amylase mixture	α-amylase	0.05% to 0.5% of α-amylase based on total weight of the dry food ingredients	No	No	U.S. 6,926,917 B2	[61]
Amino acids						
Amino acid(s)	L-phenylalanine, L-tyrosine, L-tryptophan, L-methionine, L-arginine, L-isoleucine, L-leucine, L-serine, and combinations thereof	0.001 to 0.8% based on total weight of the dry dog food	Yes	No	U.S. 4,282,254	[41]
Amino acid	L-lysine	0.1 to 500 mM	No	No	U.S. 4,267,195	[40]
Amino acid(s)	L-alanine, aspartic acid, L-asparagine, L-arginine, L-cysteine, glutamic acid, L-glycine, L-glutamine, L-histidine, L-isoleucine, L-leucine, L-lysine, L-methionine, L-phenylalanine, L-proline, L-serine, L-threonine, L-tryptophan, L-tyrosine, L-valine, and combinations thereof	1 to 70% by weight of free amino acids	Yes	No	E.P. 2731449 B1	[72]
Aroma/flavor compounds (direct addition)						
Liquid aroma	Human aroma such as “sweet” and “grilled” along with traditional liquid palatability enhancer	Total palatant composition made of 1–5% liquid aroma and 95–99% traditional palatability enhancer	No	No	U.S. 2016/0309749 A1	[73]
Dry aroma	Dry food aromas including animal (beef, poultry, pork, fish), vegetable (herbs, fruits, vegetables), dairy (butter, milk, cheese) along with other palatability enhancers (PE; animal digests, animal fats, dairy products)	Weight ratio of dry aroma: PE could vary from 0.25: 99.75 to 20: 80. The palatability enhancer and dry food aroma is added at 0.25–12% w/w of pet food.	Yes	Yes	U.S. 2015/0056347 A1	[54]
Dry and liquid flavor	BBQ, pizza, beef stew and crackers, sausages and eggs, peanut butter and jelly, roast beef and potatoes	Depends on type pf flavor used	No	No	U.S. 6,379,727 B1	[74]
Lipids						
Fatty acids or combination of fats and oils	Ehanolamides with oleic acid/palmitic acid/stearic acid/linoleic acid/linoleic acid and combinations thereof or ethanolamides with beef tallow/cocoa butter/palm oil/palm stearin/palm fractions/olive oil/hydrogenated oils/lard/high oleic safflower and combinations thereof	10–1000 ppm	Yes	No	U.S. 2016/0029668 A1	[81]
Fats	Fat composition with varying compositions of C12 (dodecanoic acid): C10 (decanoic acid) and/or C14 (tetradecanoic acid): C12 (dodecanoic acid)	Fatty acid weight ratio of 0.85 to 2.5 (C12:0/C10:0) and/or 0.45 to 4.3 (C14:0/C12:0)	Yes	No	U.S. 2015/0237887 A1	[42]
Fats and oil	Animal fat (chicken, beef, pork), vegetable (crude soybean oil) and fish oil	50, 49, 1 parts by weight animal fat, vegetable oil and flavors, respectively or 48, 2, 49, 0.1 parts by weight animal fat, fish oil, vegetable oil and flavors, respectively	No	No	U.S. 3,745,023	[79]
Organic acids						
Organic acid	Ascorbic acid	Varies with composition of food	Yes	No	U.S. 2016/0316789 A1	[84]
Organic acid(s)	Fumaric acid alone or in combination with sorbic, succinic and gallic acids	0.1–2.0% based on total weight of the dry dog food	Yes	No	U.S. 2014/0154356 A1	[43]
Miscellaneous/others						
Zeolites	Clinoptilolite	0.01–4% based on total weight of the dry dog food	Yes	No	U.S. 2009/0274796 A1	[87]
Herb and spices	Essential oil of tarragon	0.001–0.005% based on total weight of the dry dog food	Yes	No	W.O. 2006/065841 A2	[86]
Licorice	Ammoniated glycyrrhizin	Varies with sucrose content of the pet food	Yes	No	U.S. 4,191,781	[85]

## Data Availability

Not Applicable.
